# Sustained Remission of Antineutrophil Cytoplasmic Antibody-Mediated Glomerulonephritis and Nephrotic Syndrome in Mixed Connective Tissue Disease

**DOI:** 10.4021/jocmr1391w

**Published:** 2013-06-21

**Authors:** Konstantin N. Konstantinov, Alexis A. Harris, Marc Barry, Glen H. Murata, Antonios H. Tzamaloukas

**Affiliations:** a Division of Rheumatology, University of New Mexico School of Medicine; USA; b Nephropath Renal Pathology, Little Rock, Arkansas; USA; c Division of Pathology, University of New Mexico School of Medicine; USA; d Division of General Medicine, University of New Mexico School of Medicine and Raymond G. Murphy VA Medical Center, Albuquerque, New Mexico, USA; e Division of Nephrology, University of New Mexico School of Medicine and Raymond G. Murphy VA Medical Center, Albuquerque, New Mexico, USA

**Keywords:** Mixed-connective tissue disease, Antineutrophil cytoplasmic antibodies, Antimyeloperoxidase, Nephrotic syndrome, Pauci-immune glomerulonephritis

## Abstract

A woman diagnosed with mixed connective tissue disease (MCTD) developed an anti-myeloperoxidase (MPO) antineutrophil cytoplasmic antibody (ANCA) and nephrotic syndrome with normal serum creatinine. Percutaneous kidney biopsy showed pauci-immune glomerulonephritis with superimposed immune complex deposition. After treatment with cyclophophamide and prednisone, proteinuria decreased progressively to a level of 0.4 g/g creatinine, ANCA became undetectable, while serum creatinine remained normal seven years after the beginning of treatment. Sustained remission of nephrotic proteinuria with preserved renal function may follow treatment of ANCA-mediated disease developing in patients with MCTD.

## Introduction

Mixed connective tissue disease (MCTD) is an overlap syndrome characterized by anti-U1-RNP antibodies and clinical features of systemic lupus erythematosus, scleroderma, and polymyositis [1]. Renal involvement occurs in as high as 40% in adult [2] and 47% in pediatric [3] cases. Membranous nephropathy is the most common renal histological picture. Mesangial proliferative glomerulonephritis and membrano-proliferative glomerulonephritis are less frequent [2, 4-6].

Infrequent types of renal histology in MCTD include intimal hyperplasia of the renal arterioles associated with scleroderma-type renal crisis [7] and pauci-immune glomerulonephritis, along with other features of vasculitis, following development of positive antineutrophil cytoplasmic antibody -ANCA- [8]. We report a patient with MCTD who developed anti- myeloperoxidase (MPO) ANCA positivity, pauci-immune glomerulonephritis with superimposed immune complex mediated disease, and nephrotic syndrome. Prolonged remission followed treatment directed against ANCA-associated disease.

## Case Report

A 42-year-old woman with past history of hypothyroidism, iron deficiency anemia and nasal sinusitis developed Raynaud’s phenomenon, swollen fingers and hands, esophageal dysfunction, acrosclerosis and trigeminal neuropathy. Persistently high anti-U1- RNP titers confirmed the diagnosis of MCTD. Serum rheumatoid factor and antibodies to SS-A were also elevated. Initial serum creatinine was 0.8 mg/dL and urinalysis revealed trace protein. Her medications included levothyroxine, acetaminophen and lisinopril. She did not use non-steroidal anti-inflammatory drugs. Subsequently, after being clinically stable for years, the patient developed MPO-ANCA positivity and her proteinuria increased progressively to a level of 11 g/24 h. Antibodies against PR3, dsDNA, and SSB were negative. In March 2006, she had a percutaneous kidney biopsy.

At the time of the kidney biopsy, physical findings included slim build, blood pressure of 95/60 mmHg, radial skin folding around the mouth, impaired facial muscle mobility and joint fullness without tenderness or limitation of movement. Edema was absent and the rest of the clinical examination was unremarkable. Laboratory values included erythrocyte sedimentation rate 95 mm/hr; blood hematocrit 36 vol%, hemoglobin 12 g/dL, white blood cells 6.3 K/mm^3^ and platelets 272 K/mm^3^; serum urea nitrogen 11 mg/dL, creatinine 0.9 mg/dL, and albumin 2.9 g/dL; and urine microscopy with 3 white cells and 140 red cells per high power field. No paraprotein was found in serum or urine electrophoresis.

**Figure 1:**
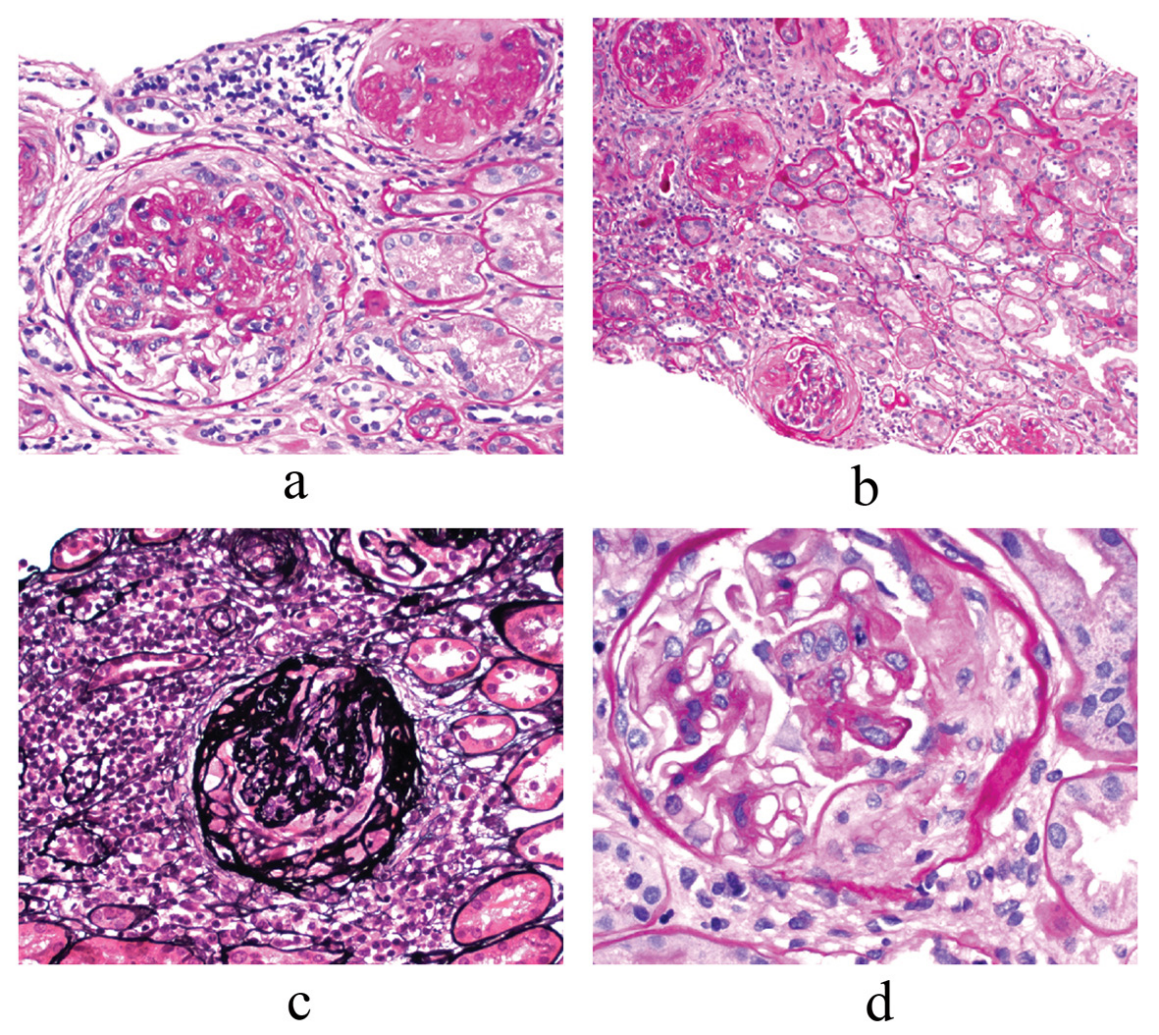
Renal Biopsy findings: Light Microscopy (LM). Focal Proliferative and sclerosing glomerulonephritis with ten percent fibrous crescents, a) Glomerulus with fibro-cellular crescent and one globally sclerotic glomerulus - 200 ×; b) Globally and segmentally sclerosed glomeruli - 40 ×; c) Jones’ stain- glomerulus with fibro-cellular crescent - 200 ×; d) Glomerulus with fibro-cellular crescent and some segmental proliferation- 400 ×.

**Figure 2:**
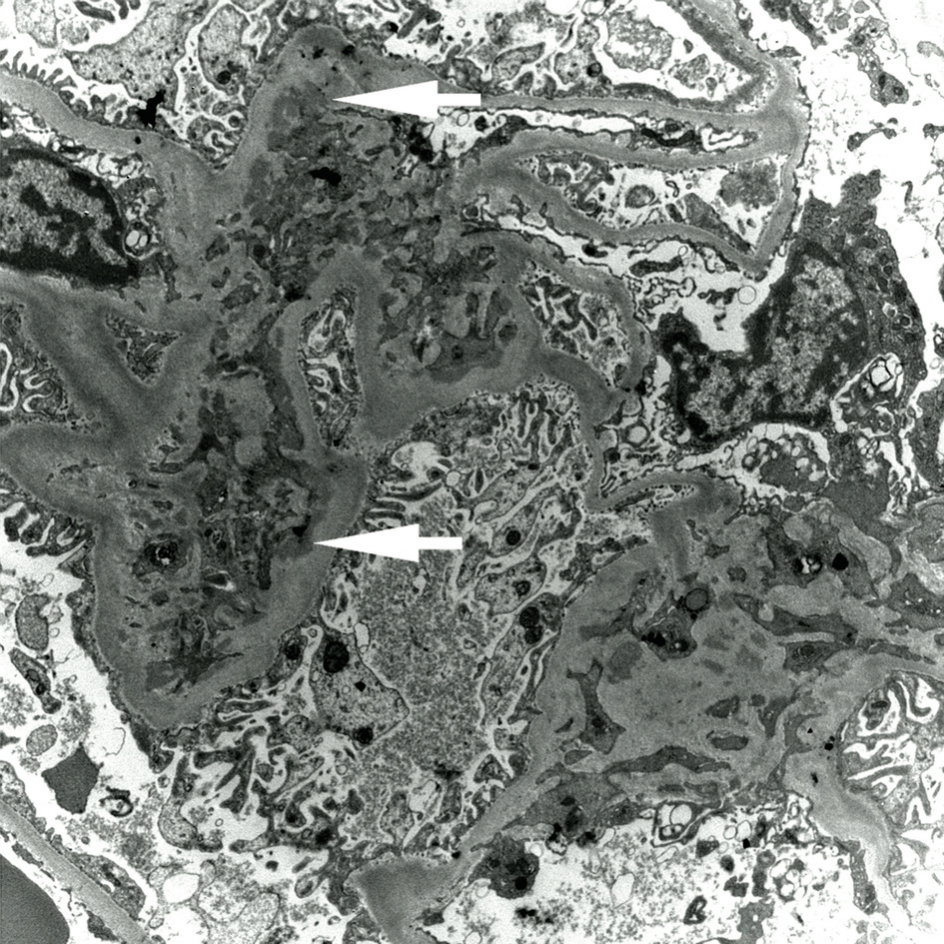
Electron Micrograph (EM). Scattered mesangial immune complex electron dense deposits (marked with white arrows), rare sub-epithelial deposit.

Renal biopsy revealed focal proliferative and sclerosing glomerulonephritis with ten percent fibrocellular crescents (light microscopy) (Fig. 1, a-d) Immunofluoressence showed scanty mesangial IgG staining and prominent granular IgG staining in the interstitium. Electron microscopy revealed rare sub-epithelial deposits and scattered mesangial deposits (Fig. 2). The histological diagnosis was pauci-immune glomerulonephritis with rare mesangial immune deposits indicative of superimposed immune-complex deposition.

She was treated with monthly intravenous cyclophosphamide (750 mg/m^2^) for 12 months and tapering dose of oral prednisone. Table 1 shows the evolution of the laboratory values. Statistical comparisons between the four periods of care shown in this table were performed by computing the 95% confidence intervals of each parameter. Serum ANA and anti-U1-RNP titers have remained elevated throughout. Serum MPO antibody decreased after treatment. Its last two measurements were within the normal range. C_4_ serum complement levels were below normal in the pre-treatment and treatment periods and increased to normal levels in the post-treatment period.

**Table 1:** Abnormal Serologies, Proteinuria and Serum Creatinine in the Patient of This Report

Laboratory value	Normal Range	1st Period	2nd Period	3rd Period	4th Period
Serum ANA titer	< 1:40	> 1:2,560	> 1:2,560	≥ 1:2,560	≥ 1:2,560
Serum Anti-U1-RNP titer	< 1:40	> 1:512	> 1:512	> 1:512	> 1:512
Serum MPO ANCA, u/L, Mean ± SD	0 - 99	35.1 ± 18.6	789	317.0 ± 306.3	173.6 ± 91.8
95% CI		-131.9-202.2		-170.4-804.3	59.6 - 287.6
Serum C_4_ complement, mg/dL, Mean ± SD	16 - 47	10.5 ± 1.2	10.0 ± 1.2	11.4 ± 1.7	17.0 ± 1.7
95% CI		7.4 - 13.5	8.9 - 11.1	10.1 - 12.7	15.5 - 18.4
Serum C_3_ complement, mg/dL, Mean ± SD	88 - 201	83.7 ± 9.1	108.3 ± 15.8	124.6 ± 12.6	129.3 ± 10.6
95% CI		60.9 - 106.4	93.6 - 122.9	114.9 - 134.2	120.3 - 138.2
Serum CRP, mg/dL, Mean ± SD	0 - 1	1.5 ± 1.0	-	1.0 ± 0.6	0.4 ± 0.2
95% CI		-0.9-3.92		0.1 - 2.0	0.1 - 0.8
ESR, mm/hr, Mean ± SD	< 20	84 ± 13	-	40	24 ± 10
95% CI		-30-198			1 - 48
Serum creatinine, mg/dL, Mean ± SD	0.66 - 1.25	0.75 ± 0.06	0.99 ± 0.16	1.00 ± 0.20	0.98 ± 0.06
95% CI		0.66 - 0.84	0.85 - 1.12	0.82 - 1.18	0.93 - 1.02
Urine protein/creatinine, gr/gr, Mean ± SD	< 0.1	1.2 ± 1.0	7.6 ± 2.3	6.4 ± 2.9	1.6 ± 1.0
95% CI		-0.3-2.8	6.1 - 9.0	4.5 - 8.3	0.9 - 2.3

1st Period: From the MCTD diagnosis until the development of the nephrotic syndrome (3 years); 2nd Period: Nephrotic syndrome prior to treatment (5 months); 3rd Period: Treatment with cyclophosphamide (1 year); 4th Period: Post-treatment follow-up (6 years). CRP: C-reactive protein; ESR: erythrocyte sedimentation rate; SD: standard deviation; 95% CI: 95% confidence interval. Large confidence intervals with negative lower limits were computed in periods with two or three measurements.

The most important findings of Table 1 are those of renal function. Serum creatinine rose slightly after development of nephrotic syndrome, but has not changed in the past seven years. Its last measurement was 0.9 mg/dL (estimated glomerular filtration rate > 60 mL/min). Proteinuria has returned to its baseline levels. In her last clinic visit, urine protein-to-creatinine ratio was 0.4 gr/gr, serum albumin was 3.9 g/dL and hematuria had disappeared. She is maintained on mycophenolate 500 mg twice daily and lisinopril. She has minimal complaints.

## Discussion

The main features of this case presentation are the sustained remission of the renal disease after biopsy-guided treatment and the finding in the renal biopsy of features suggesting superimposition of immune complex-mediated disease on pauci-immune glomerulonephritis. Development of ANCA positivity during the course of MCTD, although rare, is important because it raises the question whether ANCA-associated pathology requiring additional therapeutic interventions has also developed. Biopsy of a clinically involved organ is required to confirm the diagnosis of ANCA-associated disease because MCTD is often the cause of different types of pathology, treated potentially by different medications, in the same organs. The kidneys are one of the target organs of ANCA-associated pathology. The common forms of renal involvement in MCTD are immune complex mediated (membranous nephropathy, membranoproliferative glomerulonephritis).

We found another seven published reports of ANCA formation and evidence of renal disease in patients with MCTD [8-14]. Including the patient of this report, all eight patients were women. Age at diagnosis of ANCA positivity varied between 38 and 68 years. Six patients had MPO-ANCA [8, 10-13] (this report), one had PR3-ANCA [9], and one patient had both MPO and PR3-ANCA [14]. These patients exhibited various patterns of other autoantibodies. Serum complement levels, not reported in two patients [9, 10], were depressed in three [11, 13] (this report) and normal in the remaining three patients [8, 12, 14].

Table 2 shows renal manifestations, treatment and outcome of MCTD patients with ANCA positivity. Proteinuria and hematuria were universal. Serum creatinine, not reported in one patient [9], was normal in two [14] (this report) and elevated in the remaining five patients [8, 10-13]. Renal histology was not available in two patients [11, 14]. Among the remaining six patients, one had atherosclerosis of the renal arteries but no glomerulonephritis [9] and the other five patients had pauci-immune glomerulonephritis [8, 10, 12, 13] (this report). Vasculitis treatment (cyclophosphamide and methylprednisolone) was used for patients with pauci-immune glomerulonephritis, while the patient with atherosclerosis received lipid-lowering treatment. Two patients died during hospitalization, one from pulmonary hemorrhage and one from sepsis. Clinical, renal and serological features of the remaining patients improved with treatment.

**Table 2:** Renal Manifestations, Treatment and Outcome in MCTD Patients With ANCA Positivity

Reference	Renal Function and Urinary Findings	Renal Histology	Treatment	Follow-up	Outcome
8	S. creatinine 1.5 mg/dL Proteinuria 0.77 g/day Hematuria > 100/HPF	Pauci-immune focal necrotizing GN	Methyl-prednisolone, cyclophosphamide	< 1 month	Improved proteinuria and serology
9	S. creatinine NR Proteinuria NR Hematuria NR	Atherosclerosis No GN	Prednisolone Probucol Aspirin Sodium valproate	1 month	Improved clinical picture and serology
10	S. creatinine 4.08 mg/dL Proteinuria 6.5 g/day Hematuria NR	Fibrocellular crescents Pauci-immune necrotizing GN	Methyl-prednisolone Cyclophosphamide Azathioprine	14 months	Complete Remission
11	S. creatinine 6.0 mg/dL Proteinuria1+ Hematuria 3+	Biopsy not done	Methyl-prednisolone Hemodiafiltration	16 days	Died from pulmonary alveolar hemorrhage
12	S. creatinine 4.0 mg/dL Proteinuria 2+ Hematuria Present	Crescentic pauci-immune GN	Methyl-prednisolone Cyclophosphamide	?	Improved renal function
13	S. creatinine 2.49 mg/dL Proteinuria 1.7 g/day Hematuria Present	Fibrocellular crescents Pauci-immune GN plus immune complexes	Prednisolone	116 days	Died from sepsis (colonic perforation)
14	S. creatinine 0.5 mg/dL Proteinuria +/- Hematuria 0-1/HPF	Biopsy not done	Methyl-prednisolone Azat hioprine	42 days	Improved serology
This report	S. creatinine 0.9 mg/dL Proteinuria 11.0 g/day Hematuria 140/HPF	Fibrocellular crescents Pauci-immune GN plus immune complexes	Cyclophosphamide Prednisone	6 years	Improved clinical picture, proteinuria, serology, normal eGFR

S: serum; NR: not reported; GN: glomerulonephritis; HPF: high power field; eGFR: glomerular filtration rate estimated by the modification in diet in renal disease (MDRD) formula.

Follow-up of the other patients in Table 2 was short-term. Only one patient had a follow-up, at 14 months, longer than one year [10]. The sustained improvement of clinical picture, proteinuria and serologies after treatment of vasculitis in our patient, while her renal function remains within the normal range in six years of follow-up, provides evidence that treatment directed against documented ANCA-associated disease can produce long-term benefits in patients with MCTD.

Superimposition of immune complex deposition on ANCA-associated glomerulonephritis was the second important feature of our patient. Presences of small amounts of immunoglobulin in the mesangium and immune complex deposition on the electron microscopic picture were also documented in another MCTD patient diagnosed with pauci-immune glomerulonephritis [13]. The concept of “dual glomerulopathy” is based on the observation of histological features of pauci-immune glomerulonephritis combined with immune complex deposition the magnitude of which cannot explain the extent of the renal histological damage [15]. Superimposition of immune complexes was found in 54% of 126 cases with pauci-immune glomerulonephritis. Clinical findings suggested that the immune complex deposition worsened the severity of the renal lesion [15].

The superimposition of immune complex deposition on ANCA-associated renal disease in our patient could be the cause of two features that are uncommon in pauci-immune glomerulonephritis, low serum complement levels and nephrotic proteinuria. Serum complement levels were low in only three patients of Table 2. Our patient and the other patient with dual glomerulopathy [13] had low serum complement, while renal histology was not available in the other patient with hypocomplementemia [11]. Superimposition of immune complex deposition worsens the proteinuria in patients with pauci-immune glomerulonephritis [16]. Global and/or segmental glomerular sclerosis, another feature of our patient’s biopsy (Fig. 1), is also associated with nephrotic proteinuria [17]. Nephrotic syndrome is common in MCTD with membranous nephropathy [2], but not in pauci-immune glomerulonephritis. Nephrotic proteinuria was noticed in another patient of Table 2 [10]. After treatment of ANCA-associated disease, complete remission of the nephrotic syndrome was noted in the other patient [10], while our patient had in incomplete, but sustained, remission.

The development of ANCA positivity in patients with MCTD should trigger a systematic search for clinical manifestations of ANCA-mediated disease. These manifestations should guide biopsies of involved organs. Proteinuria, abnormal urine sediment and rise in serum creatinine are indications for kidney biopsy. Treatment directed towards ANCA-associated disease should be instituted if the renal biopsy confirms this diagnosis. Sustained remission may follow.
